# Supplementary material for: Giantin-knockout models reveal a feedback loop between Golgi function and glycosyltransferase expression
Source: J Cell Sci. 2017 Dec 15;130(24):4132–43. doi: 10.1242/jcs.212308 (PMC5769581; doi:10.1242/jcs.212308)
Supplement: Supplementary information [file joces-130-212308-s1.pdf]

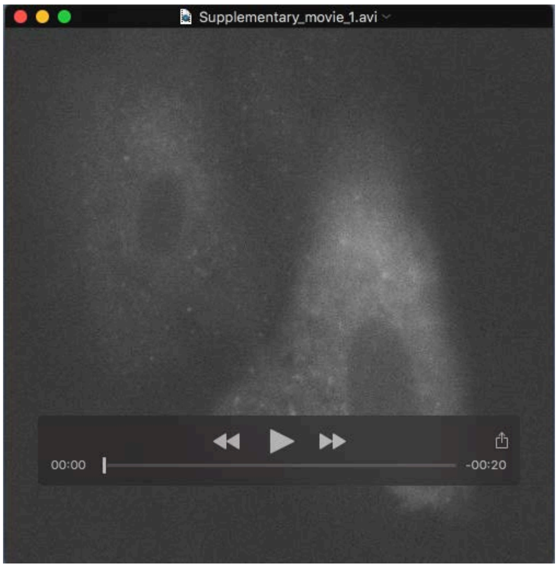

Movie S1 relating to Figure 2: RUSH trafficking in WT cells. WT cells transfected with Str-Kdel/ManII-SBP-EGFP were treated with biotin at T0 and imaged live as a single plane at 4 frames per minute.

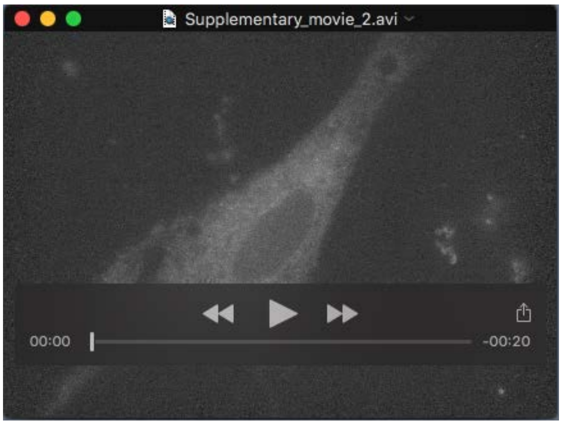

Movie S2 relating to Figure 2: RUSH trafficking in KO cells. Giantin KO cells transfected with Str-Kdel/ManII-SBP-EGFP were treated with biotin at T0 and imaged live as a single plane at 4 frames per minute.

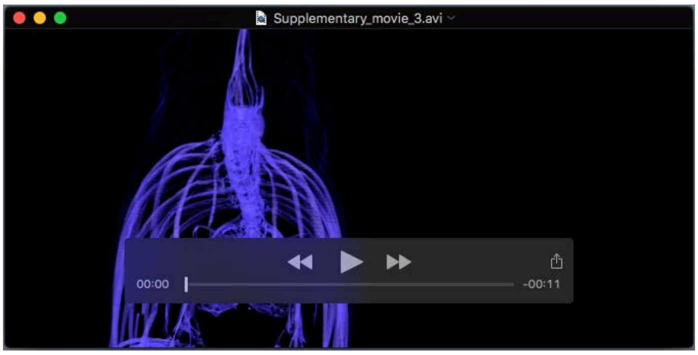

Movie S3 relating to Figure 5: *golgb1*$^{wt/wt}$ sibling fly-through showing absence of ectopic deposits around the spine. 4 µm voxel size microCT scan.

Journal of Cell Science • Supplementary information

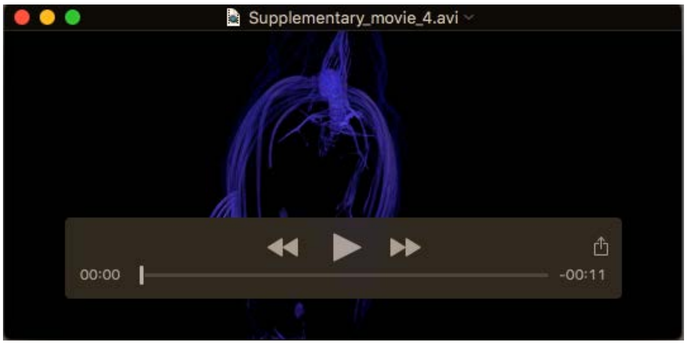

Movie S4 relating to Figure 5: *golgb1*$^{Q2948X/Q2948X}$ mutant fly-through showing ectopic deposits around the spine. 4 µm voxel size microCT scan.

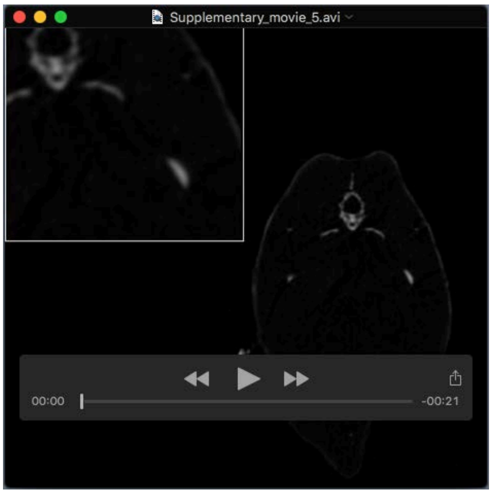

Movie S5 relating to Figure 5: *golgb1*$^{wt/wt}$ sibling showing axial microCT slices through the spine. 21.8 µm voxel size.

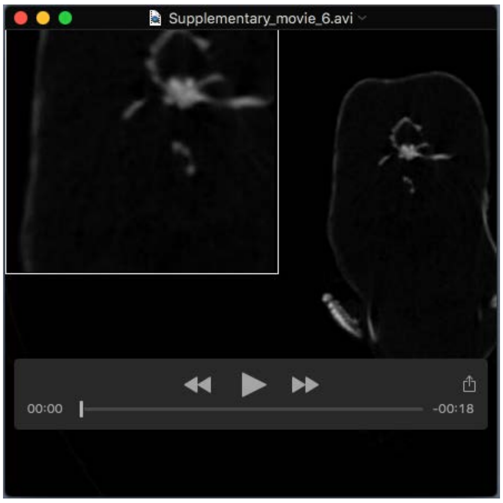

Movie S6 relating to Figure 5: *golgb1*$^{Q2948X/Q2948X}$ mutant showing axial microCT slices with calcified deposits in the spine. 21.8 µm voxel size.

Table S1 relating to Table 1: RNAseq results of pairwise comparison of wild-type and giantin KO cells. The first tab shows all data, the second shows those genes that have changed from than 2-fold, the third, those that have changed more than 3-fold.

Click here to Download Table S1

Table S1: Excel spreadsheet of RNAseq data

Column number	Column name	Example	Description
1	Tested id	A1BG	A unique identifier describing the transcript, gene, primary transcript, or CDS being tested
2	gene	A1BG	The gene_name(s) or gene_id(s) being tested
3	locus	chr19:58346805--58362848	Genomic coordinates for easy browsing to the genes or transcripts being tested.
4	sample 1	RPE--WT	Label of the first sample
5	sample 2	RPE--giantin_KO	Label of the second sample
6	Test status	OK	Can be one of OK (test successful), NOTEST (not enough alignments for testing), LOWDATA (too complex or shallowly sequenced), HIDATA (too many fragments in locus), or FAIL, when an ill--conditioned covariance matrix or other numerical exception prevents testing.
7	FPKMx	1.78215	FPKM of the gene in sample x
8	FPKMy	2.77931	FPKM of the gene in sample y
10	Test stat	1.16871	The value of the test statistic used to compute significance of the observed change in FPKM
11	p	0.0513	The uncorrected p--value of the test statistic
12	q	0.10281	The FDR--adjusted p--value of the test statistic
13	significant	no	Can be either “yes” or “no”, depending on whether p is greater than the FDR after Benjamini-Hochberg correction for multiple--testing

Journal of Cell Science • Supplementary information

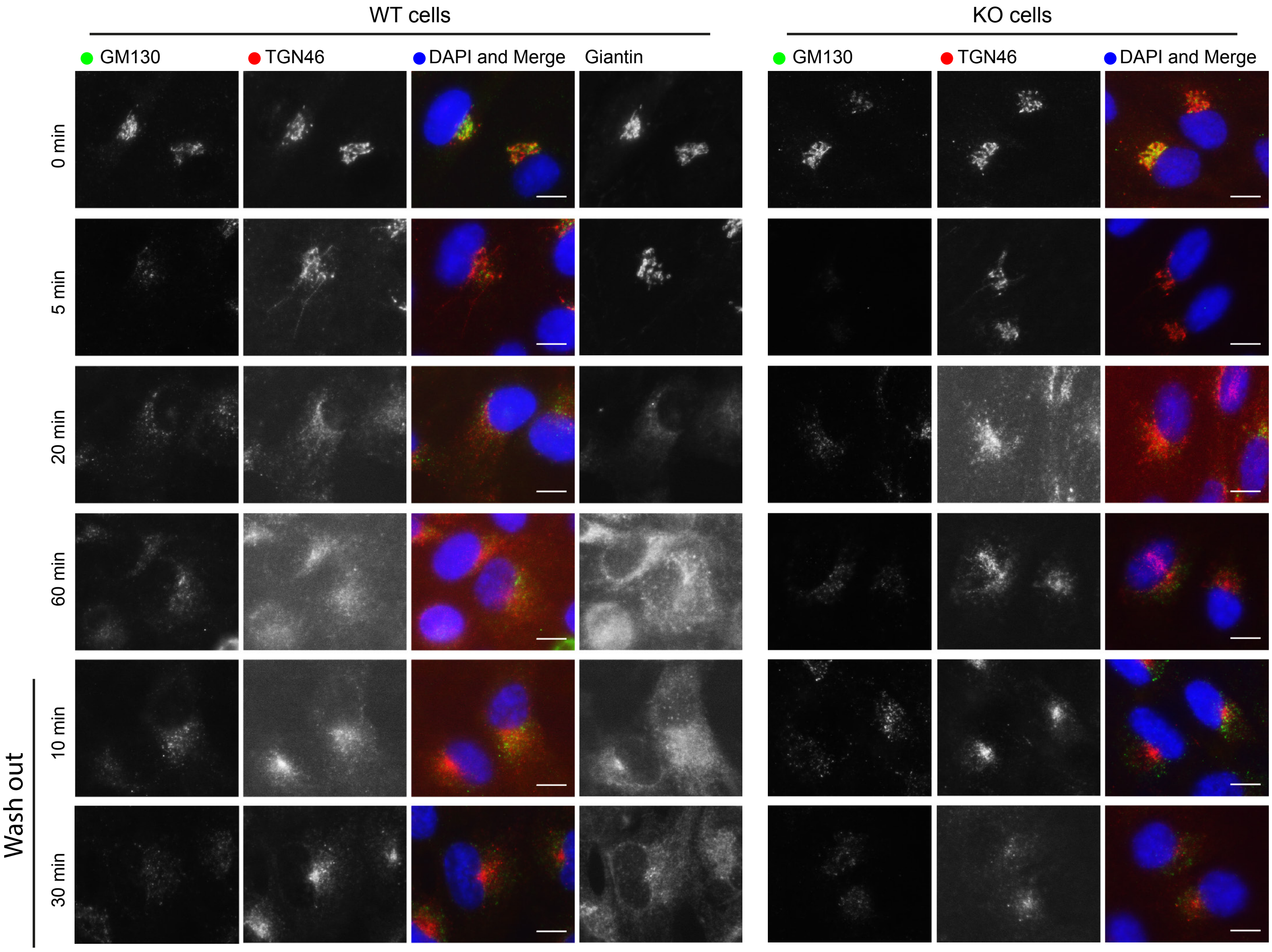

Figure S1 relating to Figure 3: Brefeldin A treatment of KO cells. Representative maximum projection images of WT and KO cells treated with 5 µm Brefeldin A for time indicated and immuno-labelled for cis-(GM130) and trans-(TGN46) Golgi markers. In wash out panels, cells were incubated in brefeldin A for 1 hour then washed 3x and left in growth media at 37°C for time indicated. Scale bars 10 µm.

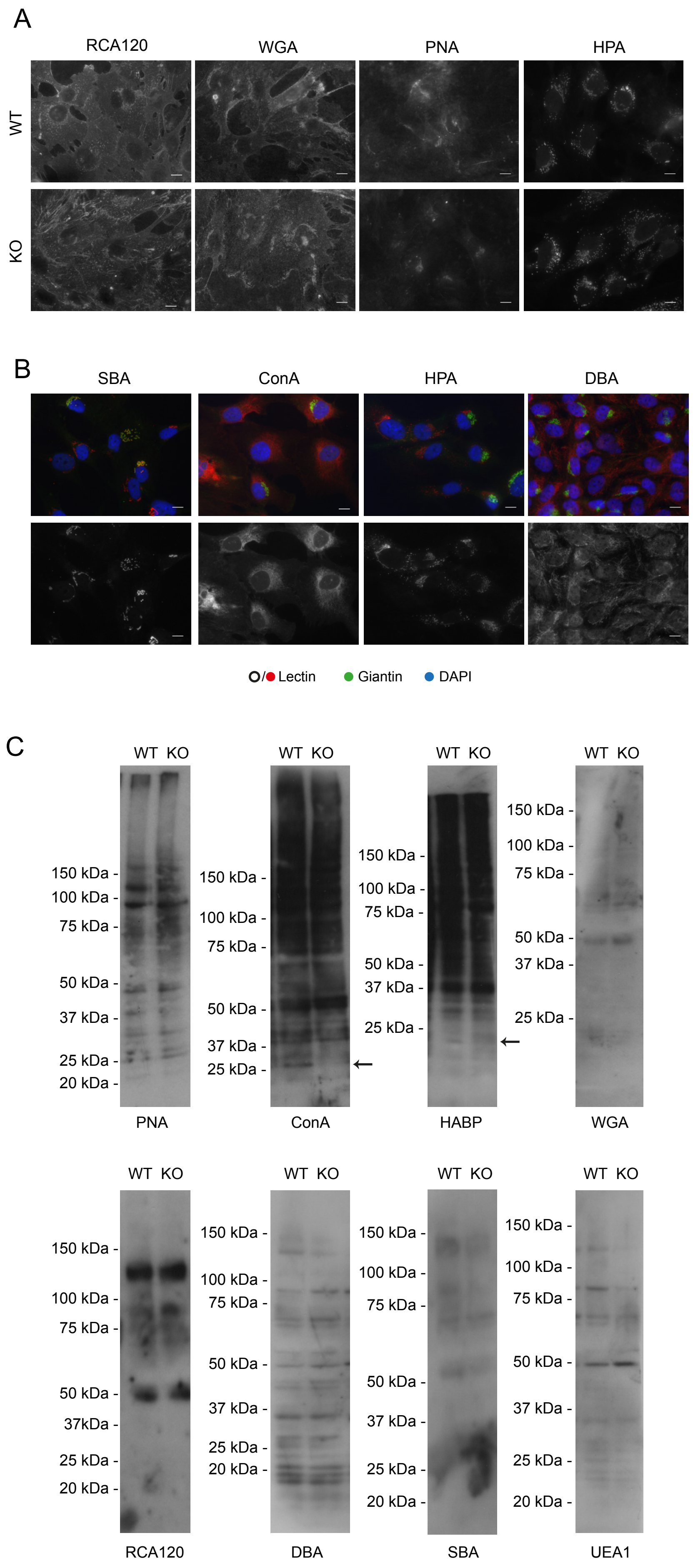
A
RCA120
WGA
PNA
HPA
WT
KO
B
SBA
ConA
HPA
DBA
Lectin
Giantin
DAPI
C
WT KO
150 kDa -
100 kDa -
75 kDa -
50 kDa -
37 kDa -
25 kDa -
20 kDa -
PNA
ConA
HABP
WGA
RCA120
DBA
SBA
UEA1

Figure S2 relating to Figure 4: Lectin labelling in giantin KO cells. Lectin labelling of WT and KO cells without permeabilisation (A) and mixed populations of permeabilised WT and KO cells (B). Images are maximum projections. Scale bars 10 µm. C. Western blots of WT and KO cell lysates probed with lectins. Arrows highlight missing bands.

Journal of Cell Science • Supplementary information

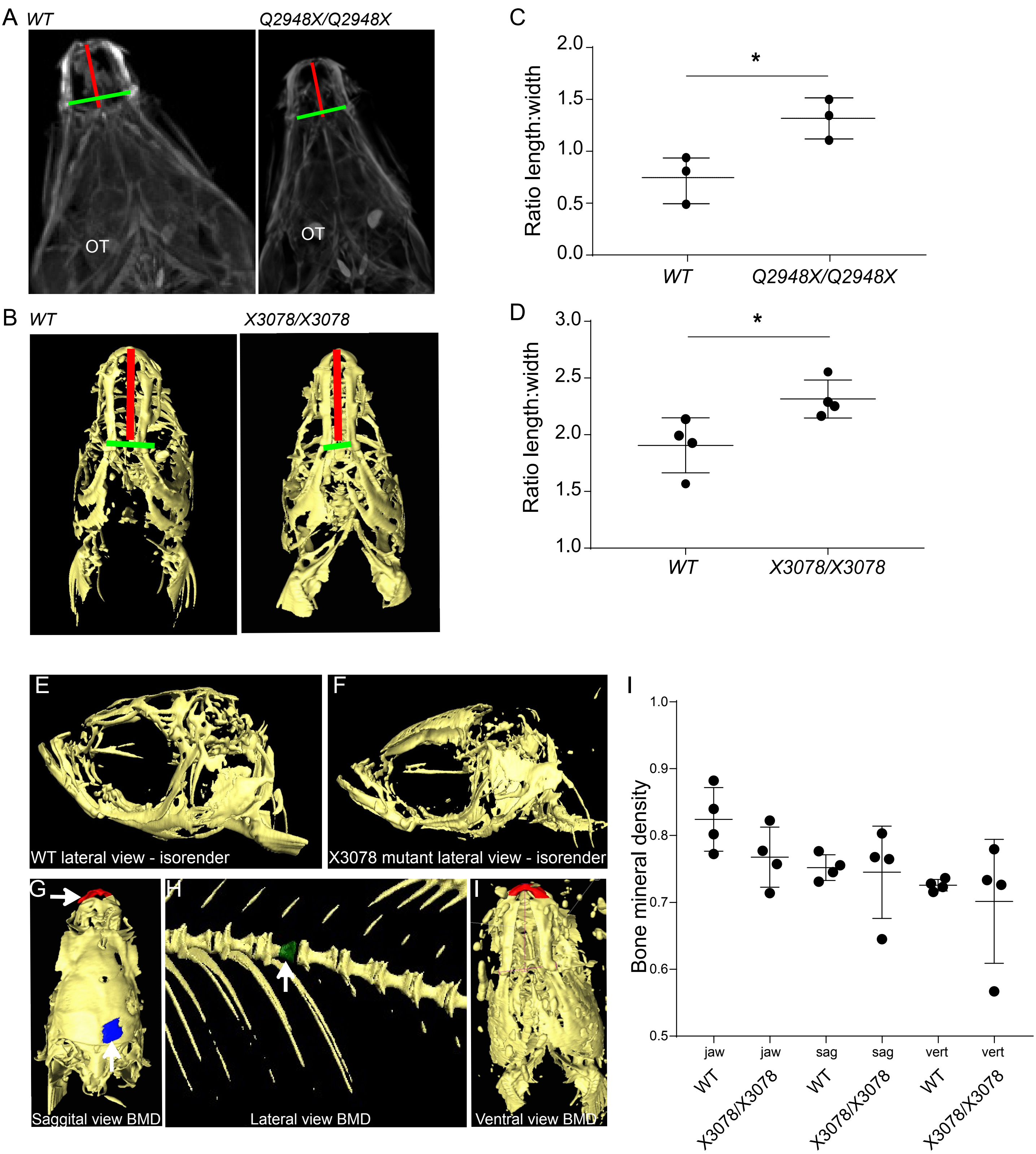

Journal of Cell Science • Supplementary information

Figure S3 relating to Figure 5: (A) Ventral jaw element views in 8 months old WT and *golgb1*$^{Q2948X/Q2948X}$ zebrafish, and (B) in 10-month *golgb1*$^{X3078/X3078}$ mutant and WT zebrafish derived from micro CT images. The relative distance from the anterior most point of the lower jaw to the mid-point between the jaw joints and the width between joints is demarked by the red and green line respectively and the ratio between these is calculated in C and D. (E, F) Ventral isorenders of the (E) WT and (F) *golgb1*$^{X3078/X3078}$ mutants. (G-I) Red and blue patches highlight regions on isosurface renders of CT images used for calculation of BMD from a (G) saggital view of the skull, (H) lateral view of the vertebral column and (I) ventral view of the jaw. (J) Quantification of BMD shows no significant differences between WT and mutants in jaw, saggital, or vertebral measurements. Data were analysed using an unpaired t-test; p value: *= <0.05, mean and standard deviation). Data was taken from between 15 and 30 sections per fish (details in methods).
